# REFLECTIVE THINKING OF THE CSMP APP CO-DESIGN AND ALPHA-TESTING PROCESS

**DOI:** 10.1093/geroni/igz038.2013

**Published:** 2019-11-08

**Authors:** Kexin Yu, Shinyi Wu, Mandong Liu, Haojun Jiang, Iris Chi

**Affiliations:** 1 University of Southern California, Los Angeles, California, United States; 2 Nanjing Agricultural University, Nanjing, Jiangsu, China (People’s Republic)

## Abstract

Time and transportation barriers prevented caregivers with busy schedules to attend the CSMP. Developing a CSMP mobile application (app) and testing its feasibility was hypothesized to increase the accessibility of the CSMP to caregivers. Applying a user-participatory approach, seven caregivers who attended the CSMP in-person intervention participated in a 2-hour co-design session. A CSMP app prototype was developed based on the co-design interview results. The same seven caregivers and another five caregivers who were naïve to the CSMP intervention participated in a 2-hour alpha testing interview to provide feedback to the app prototype. Compared to co-design, participants were able to provide more constructive suggestions regarding app design when the app prototype was shown. The user participatory approach is a dialogue between researchers’ knowledge framework and users’ lived experience. Both agreement and disagreement regarding app design were identified, yet how to address the discrepancy has not been discussed in the literature.

